# Impact of multidrug resistance on outcomes in hematologic cancer patients with bacterial bloodstream infections

**DOI:** 10.1038/s41598-024-66524-w

**Published:** 2024-07-07

**Authors:** Ki-Ho Park, Ye Ji Jung, Hyun Jung Lee, Hong Jun Kim, Chi Hoon Maeng, Sun Kyung Baek, Jae Joon Han, Woojae Jeon, Dong Youn Kim, Yu-Mi Lee, Mi Suk Lee

**Affiliations:** 1 Department of Infectious Diseases, Kyung Hee University College of Medicine, Seoul, Republic of Korea; 2 Department of Hematology and Medical Oncology, Kyung Hee University College of Medicine, 26 Kyungheedae-ro, Dongdaemun-gu, Seoul, 02447 Republic of Korea

**Keywords:** Hematological cancer, Bloodstream infection, Multidrug-resistant organisms, Outcome, Mortality, Haematological cancer, Infectious diseases

## Abstract

Despite the improved outcomes in patients with hematological malignancies, infections caused by multidrug-resistant organisms (MDROs) pose a new threat to these patients. We retrospectively reviewed the patients with hematological cancer and bacterial bloodstream infections (BSIs) at a tertiary hospital between 2003 and 2022 to assess the impact of MDROs on outcomes. Among 328 BSIs, 81 (24.7%) were caused by MDROs. MDRO rates increased from 10.3% (2003–2007) to 39.7% (2018–2022) (*P* < 0.001). The 30-day mortality rate was 25.0%, which was significantly higher in MDRO-infected patients than in non-MDRO-infected patients (48.1 vs. 17.4%; *P* < 0.001). The observed trend was more pronounced in patients with newly diagnosed diseases and relapsed/refractory disease but less prominent in patients in complete remission. Among MDROs, carbapenem-resistant Gram-negative bacteria exhibited the highest mortality, followed by vancomycin-resistant enterococci, methicillin-resistant *Staphylococcus aureus*, and extended-spectrum β-lactamase-producing *Enterobacteriaceae*. Multivariate analysis identified independent risk factors for 30-day mortality as age ≥ 65 years, newly diagnosed disease, relapsed/refractory disease, MDROs, polymicrobial infection, CRP ≥ 20 mg/L, and inappropriate initial antibiotic therapy. In conclusion, MDROs contribute to adverse outcomes in patients with hematological cancer and bacterial BSIs, with effects varying based on the underlying disease status and causative pathogens. Appropriate initial antibiotic therapy may improve patient outcomes.

## Introduction

Survival outcomes in patients with hematological malignancies have substantially improved owing to recent advances in therapy, and the causes of death are changing. Non-cancer-related deaths are now more significant than cancer-related deaths, with infections being important contributors^1^. Patients with hematological malignancies are considered part of the immunocompromised group owing to the disease, host factors, and treatment-related factors such as chemotherapy, steroids, and severe/prolonged neutropenia^2^. Bloodstream infections (BSIs) are the most severe infection, occurring in approximately one-third of febrile episodes in patients with hematological malignancies^3,4^. BSIs disrupt chemotherapy, and the overall 30-day mortality rates are high, ranging between 12 and 50%^5–7^.

Antimicrobial resistance has increased in recent decades, posing a new threat worldwide. Multidrug-resistant organisms (MDROs) are frequently resistant to three or more antibiotics^8^. Carbapenem-resistant Gram-negative bacteria (GNB), extended-spectrum β-lactamase (ESBL)-producing *Enterobacteriaceae*, vancomycin-resistant enterococci (VRE), and methicillin-resistant *Staphylococcus aureus* (MRSA) are highly resistant organisms that deserve special attention in healthcare facilities^9^. MDROs are increasingly implicated in bacteremia patients with hematological malignancies^7,10,11^. Recent studies have demonstrated that the clinical outcomes of hematologic cancer patients with bacterial BSIs caused by MDROs are worse than those of patients with bacterial BSIs caused by non-MDROs^7,12^.

To comprehensively assess the impact of MDROs on the outcomes of patients with hematological cancer and bacterial BSIs, we conducted a retrospective analysis spanning two decades. This study aimed to elucidate evolving trends in MDRO prevalence, associated mortality rates, and factors influencing clinical outcomes.

## Methods

### Study design and setting

This study was conducted at an 850-bed tertiary-care academic center in Seoul, South Korea. We retrospectively reviewed the charts of all bacterial BSIs that developed in adult (≥ 18 years) patients with hematological cancer between January 2003 and December 2022. This study was approved by the institutional review board of Kyung Hee University Hospital (2023–06-068). The requirement for informed consent was waived owing to the retrospective nature of the study.

### Study patients

Bacterial BSI was defined as the presence of bacterial growth in blood cultures. Clinical and microbiological assessments were performed to determine the etiological significance of the isolated pathogens. Coagulase-negative staphylococci, *Bacillus* spp., *Corynebacterium* spp., and *Cutibacterium acnes* were considered contaminants unless they were isolated from two or more separate blood culture sets. Polymicrobial BSI was defined as the detection of two or more different bacterial organisms on the first day of a BSI episode. When a patient experienced more than one episode of bacterial BSI during each admission, only the first episode for each admission was considered to avoid non-independence associated with repeated measures.

### Definitions

In several previous studies, MDROs were defined as bacteria showing resistance to at least one agent in three or more antibiotic classes^8,11,13^. However, because of inconsistencies in the antimicrobials included in the automated susceptibility test panels in our hospital over the 20-year study period, MDROs were composed of typical highly resistant organisms, including MRSA, VRE, ESBL-producing *Enterobacteriaceae*, and carbapenem-resistant GNB^9^. Carbapenem-resistant GNB were defined as *Enterobactericae*, *Pseudomonas aeruginosa,* and *Acinetobacter* species that exhibit non-susceptibility to at least one of three carbapenem antibiotics (imipenem, meropenem, and doripenem). *Stenotrophomonas maltophilia*, which is intrinsically resistant to carbapenem antibiotics, was also classified as carbapenem-resistant GNB^14^. The susceptibility of bacteria to antibiotics was determined according to the guidelines of the Clinical and Laboratory Standards Institute (CLSI)^14^. The susceptibility breakpoints for imipenem, meropenem, and doripenem in *Enterobacteriaceae* were revised from ≤ 4 to ≤ 1 mg/L in 2010. For *Pseudomonas aeruginosa*, the susceptibility breakpoints were revised from ≤ 4 to ≤ 2 mg/L in 2012. Similarly, the susceptibility breakpoints for *Acinetobacter* species were revised from ≤ 4 to ≤ 2 mg/L in 2014^14,15^. To quantify the overall comorbidity burden, we calculated Charlson Comorbidity Index scores using codes from the International Statistical Classification of Diseases and Related Health Problems, 10th Revision (ICD-10)^16,17^. The initial antibiotic therapy administered within the first 72 h was categorized as appropriate or inappropriate, depending on the results of antibiotic susceptibility testing^18^.

### Statistical analyses

Chi-square or Fisher’s exact tests were used to compare categorical variables between the two groups, as appropriate. Student’s t-test and Mann–Whitney U test were used for continuous variables with normal and non-normal distributions, respectively. The normality of the distribution was assessed using the Shapiro–Wilk test. We used a linear-by-linear association test for ordinal data. Univariate and multivariate logistic regression analyses were performed to identify independent factors associated with 30-day mortality. A multivariate logistic regression model included all significant variables *(P* ≤ 0.05) following the univariate analysis. Continuous variables that were significant predictors in the univariate analysis were dichotomized using cut-off values derived from the classification and regression tree^19^. These dichotomized variables were then included in the multivariate model. Survival was determined using the Kaplan–Meier method, and the survival curves of the two groups were compared using the log-rank test. All statistical tests were two-tailed, and a *P* value ≤ 0.05 was considered statistically significant. All analyses were performed using R statistical software (version 4.3.2; R Foundation for Statistical Computing, Vienna, Austria).

### Ethical approval and Informed consent

All procedures performed in studies involving human participants were in accordance with the ethical standards of the institutional research committee and with the 1964 Helsinki Declaration and its later amendments or comparable ethical standards. The Institutional Review Board of the Kyung Hee University Hospital approved this study (approval number 2023-06-068), and given its retrospective nature, written informed consent was waived.

## Results

### Patient characteristics

**Figure 1:**
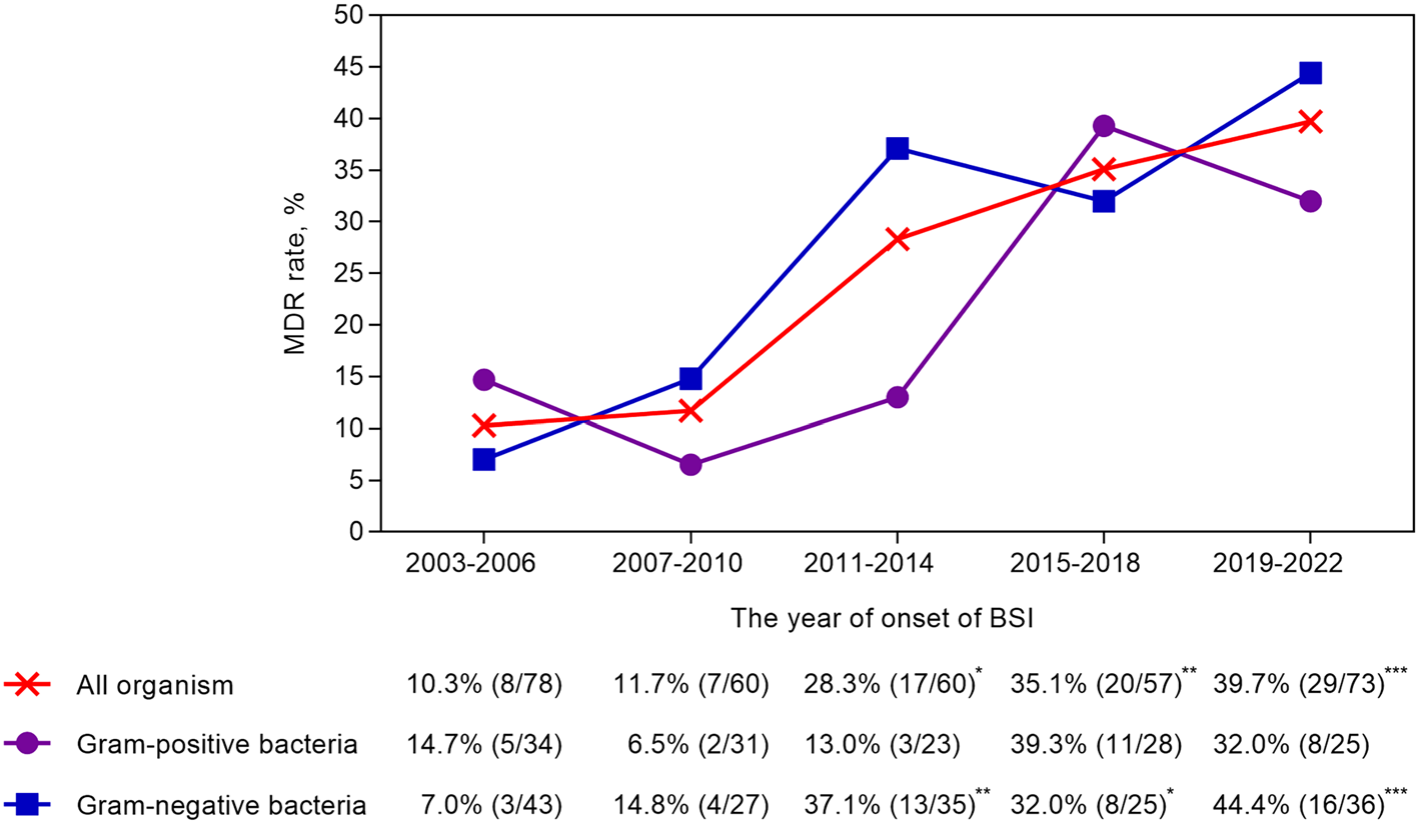
Twenty-year trends in the rates of multidrug resistance (MDR) in bacterial bloodstream infections (BSIs) in patients with hematological cancer. MDR rates during the periods of 2007–2010, 2011–2014, 2015–2018, and 2019–2022 were compared with those during the period of 2003–2006. The statistical significance was displayed using the following notation: ^*^P < 0.05, ^**^P < 0.01, and ^***^P < 0.001.

During the 20-year study period, we analyzed 328 episodes of bacterial BSIs in 228 patients with hematological malignancies. Of them, 67 (29.4%) patients experienced multiple episodes of bacteremia, ranging from 2 to 6 occurrences during the study period. Of 328 BSIs, 81 (24.7%) were caused by MRDOs. The prevalence of MDROs steadily increased from 10.3% during 2003–2007 to 39.7% during 2018–2022 (*P* < 0.001). Specifically, the prevalence of Gram-positive MDROs increased from 14.7% to 32.0% (*P* = 0.21) and the prevalence of Gram-negative MDROs increased from 7.0 to 44.4% (*P* < 0.001) (Fig. 1). Among the 328 episodes of bacterial BSIs, Gram-positive bacteria were identified as the causative agents in 141 cases (43.0%), GNB in 166 (50.6%), anaerobes in 3 (0.9%), and polymicrobial bacteria in 18 episodes (5.5%) (Table 1). Table 1 shows the details of bacterial pathogens in patients with bacterial BSIs, distinguishing between those with and without neutropenia.

**Table 1:** Organisms isolated from 328 bacterial BSI episodes in patients with hematological malignancies.

Organisms	All patients (*n* = 328)	Non-neutropenic patients (*n* = 132)	Neutropenic patients (*n* = 196)	*P*-value
Gram-positive bacteria	141 (43.0)	81 (61.4)	60 (30.6)	< 0.001
Methicillin-susceptible *S. aureus*	16 (4.9)	8 (6.1)	8 (4.1)	0.58
Methicillin-resistant *S. aureus*	11 (3.3)	7 (5.3)	4 (2.0)	0.13
Coagulase-negative staphylococci	57 (17.4)	39 (29.5)	18 (9.2)	< 0.001
*Streptococcus* species^a^	22 (6.7)	16 (12.1)	6 (3.1)	0.003
Vancomycin-susceptible enterococci	17 (5.2)	5 (3.8)	12 (6.1)	0.50
Vancomycin-resistant enterococci	18 (5.5)	6 (4.6)	12 (6.1)	0.71
Gram-negative bacteria	166 (50.6)	45 (34.1)	121 (61.8)	< 0.001
Non-ESBL-producing *Enterobactericae*	97 (29.6)	24 (18.2)	73 (37.3)	< 0.001
ESBL-producing *Enterobactericae*	23 (7.0)	8 (6.1)	15 (7.7)	0.74
Carbapenem-resistant *Enterobactericae*	4 (1.2)	0	4 (2.0)	0.15
Carbapenem-susceptible non-fermenter^b^	25 (7.6)	8 (6.1)	17 (8.7)	0.51
Carbapenem-resistant non-fermenter^c^	17 (5.2)	5 (3.7)	12 (6.1)	0.50
Anaerobes	3 (0.9)	0	3 (1.5)	0.28
Polymicrobials	18 (5.5)	6 (4.5)	12 (6.1)	0.71

The data are presented as no. (%) of patients, unless otherwise indicated.

BSI, bloodstream infection; ESBL, extended-spectrum β-lactamase.

^a^Included *Streptococcus pneumoniae* (*n* = 12), viridans streptococci (*n* = 9), and *Streptococcus agalactiae* (*n* = 1).

^b^Included *Pseudomonas aeruginosa* (*n* = 20), *Acinetobacter baumannii* (*n* = 2), *Acinetobacter lwooffii* (*n* = 2), and *Sphingomonas paucimobilis* (*n* = 1).

^c^Included *Pseudomonas aeruginosa* (*n* = 8), *Stenotrophomonas maltophilia* (*n* = 5), *and Acinetobacter baumannii* (*n* = 4).

### Baseline risk factors for MDROs

**Figure 2:**
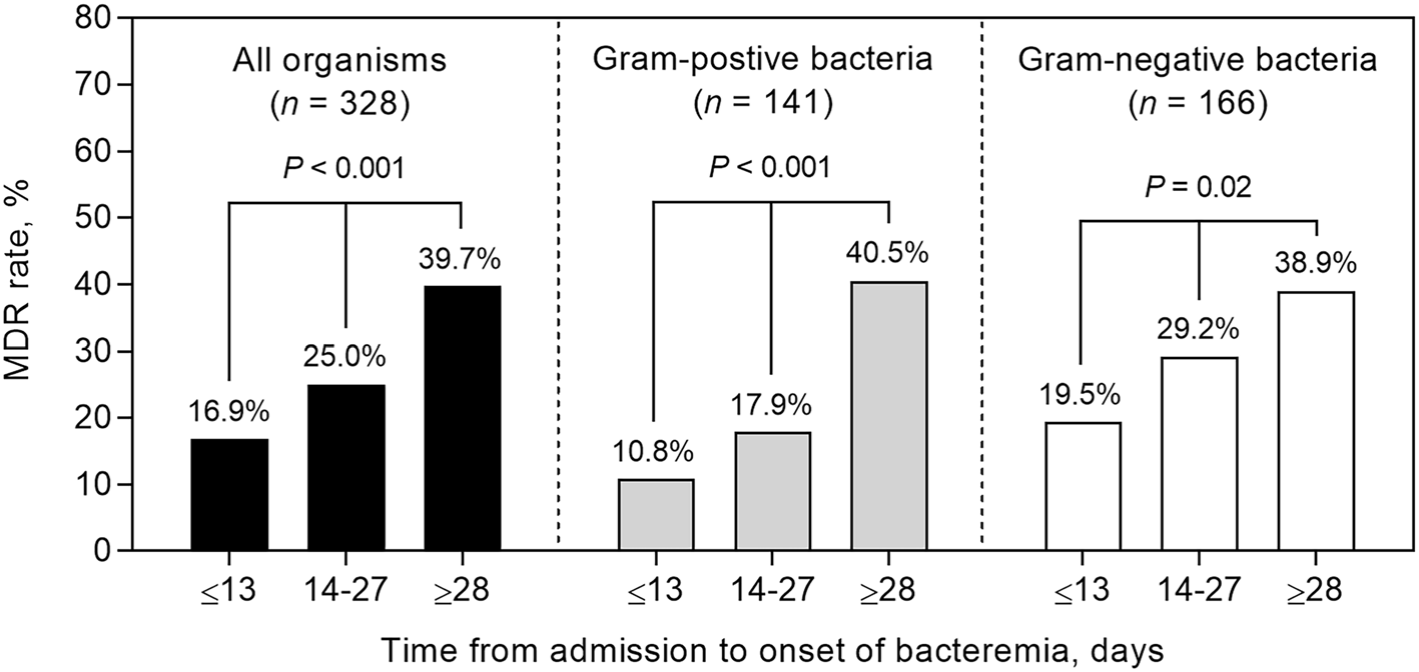
The multidrug resistance (MDR) rates according to length of hospital stay. A significant trend was observed, showing an increase in MDR rates with more extended hospital stays. The *P*-value was calculated using the linear-by-linear association test.

Table 2 shows the baseline characteristics and outcomes of 328 patients with hematologic cancer and bacterial BSIs caused by MDROs and non-MDROs. Patients infected with MDROs were more likely to have a more extended hospital stay before bacteremia onset (median 22 vs. 13 days; *P* = 0.001), lower platelet counts (median 28 vs. 44 × 10^3^/µL; *P* < 0.001), and higher C-reactive protein levels (median 16 vs. 10 mg/dL; *P* = 0.001) than those infected with non-MDROs. Patients infected with MDROs were more likely to receive inappropriate initial antibiotic therapy (42.0 vs. 13.8%, *P* < 0.001) (Table 2). There was a significant trend towards increasing MDR rate according to the length of hospital stay: 16.9% (≤ 13 days), 25.0% (14–27 days), and 39.7% (≥ 28 days) (linear-by-linear association test; *P* < 0.001) (Fig. 2). This trend was also evident among patients infected with Gram-positive cocci (10.8%, 17.9%, and 40.5%; *P* < 0.001) and Gram-negative bacilli (19.5%, 29.2%, and 38.9%; *P* = 0.02) (Fig. 2). Acute myeloid leukemia (AML) was associated with a higher rate of MDROs and these patients exhibited the longest duration of hospitalization (median, 19 days) compared with those with acute lymphoid leukemia, lymphoma, and myeloma (median, 18, 11, and 4 days, respectively).

**Table 2:** Baseline characteristics and outcomes of 328 patients with hematological cancer with bacteremia caused by multidrug-resistant organisms (MDROs) and non-MDROs.

Characteristic	All patients (*n* = 328)	Non-MDROs (*n* = 247)	MDROs (*n* = 81)	*P*-value
Age, years	61 (50–72)	61 (48–71)	61 (50–74)	0.36
Male sex	169 (51.5)	134 (54.3)	35 (43.2)	0.11
Underlying comorbidity				
Diabetes mellitus	67 (20.4)	49 (19.8)	18 (22.2)	0.76
Cerebrovascular accident	17 (5.2)	13 (5.3)	4 (4.9)	0.99
Liver cirrhosis	14 (4.3)	9 (3.6)	5 (6.2)	0.35
Congestive heart failure	10 (3.0)	7 (2.8)	3 (3.7)	0.71
Myocardial infarct	9 (2.7)	5 (2.0)	4 (4.9)	0.23
End stage renal disease	5 (1.5)	2 (0.8)	3 (3.7)	0.10
Chronic obstructive lung disease	5 (1.5)	2 (0.8)	3 (3.7)	0.10
Charlson comorbidity index score	2 (0–3)	2 (0–2)	2 (1–3)	0.009
Underlying hematologic diseases				
Acute myeloid leukemia	123 (37.5)	82 (33.2)	41 (50.6)	0.007
Acute lymphoid leukemia	26 (7.9)	20 (8.1)	6 (7.4)	0.99
Lymphoma	82 (25.0)	67 (27.1)	15 (18.5)	0.16
Multiple myeloma	62 (18.9)	52 (21.1)	10 (12.3)	0.12
Myelodysplatic syndrome	27 (8.2)	19 (7.7)	8 (9.9)	0.70
Chronic myeloid leukemia	3 (0.9)	3 (1.2)	0	0.99
Chronic lymphoid leukemia	3 (0.9)	3 (1.2)	0	0.99
Other^a^	2 (0.6)	1 (0.4)	1 (1.2)	0.43
Stage of hematological malignancy				0.16
Newly diagnosed	64 (19.5)	45 (18.2)	19 (23.5)	
In complete remission	126 (38.4)	102 (41.3)	24 (29.6)	
Relapsed/refractory	138 (42.1)	100 (40.5)	38 (46.9)	
HSCT at onset of BSI	7 (2.1)	7 (2.8)	0	0.20
Post-HSCT	14 (4.3)	10 (4.0)	4 (4.9)	0.75
Underlying conditions				
LOS until onset of bacteremia, days	14 (2–27)	13 (1–23)	22 (6–36)	0.001
ICU stay at onset of bacteremia	5 (1.5)	3 (1.2)	2 (2.5)	0.60
Presence of central venous catheters	248 (75.6)	185 (74.9)	63 (77.8)	0.71
MBI-LCBI	120 (36.6)	89 (36.0)	31 (38.3)	0.82
Laboratory data				
WBC,/µL	655 (150–3520)	800 (150–3870)	590 (170–2650)	0.63
ANC,/μL	110 (27–2124)	125 (29–2422)	72 (25–1298)	0.24
ANC < 500/μL	196 (59.8)	142 (57.5)	54 (66.7)	0.18
ANC < 100/μL	161 (49.1)	118 (47.8)	43 (53.1)	0.48
Platelet, × 10^3^/µL	39 (22–77)	44 (26–87)	28 (12–48)	< 0.001
C-relative protein, mg/dL^b^	11 (4–19)	10 (4–17)	16 (6–24)	0.001
Inappropriate initial antibiotic treatment	68 (20.7)	34 (13.8)	34 (42.0)	< 0.001
Outcomes				
7-day deaths	41 (12.5)	19 (7.7)	22 (27.2)	< 0.001
14-day deaths	61 (18.6)	30 (12.1)	31 (38.3)	< 0.001
30-day deaths	82 (25.0)	43 (17.4)	39 (48.1)	< 0.001
In-hospital mortality	107 (32.6)	60 (24.3)	47 (58.0)	< 0.001

The data are presented as no. (%) of patients or median (interquartile range), unless otherwise indicated.

ANC, absolute neutrophil count; ICU, intensive care unit; LOS, length of stay; MBI-LCBI, mucosal barrier injury laboratory-confirmed bloodstream infection; WBC, white blood cell.

^a^Included myelofibrosis (*n* = 1) and Waldenström macroglobulinemia (*n* = 1).

^b^Measured in 305 patients.

### Impact of disease status and MDROs on patient outcome

**Figure 3:**
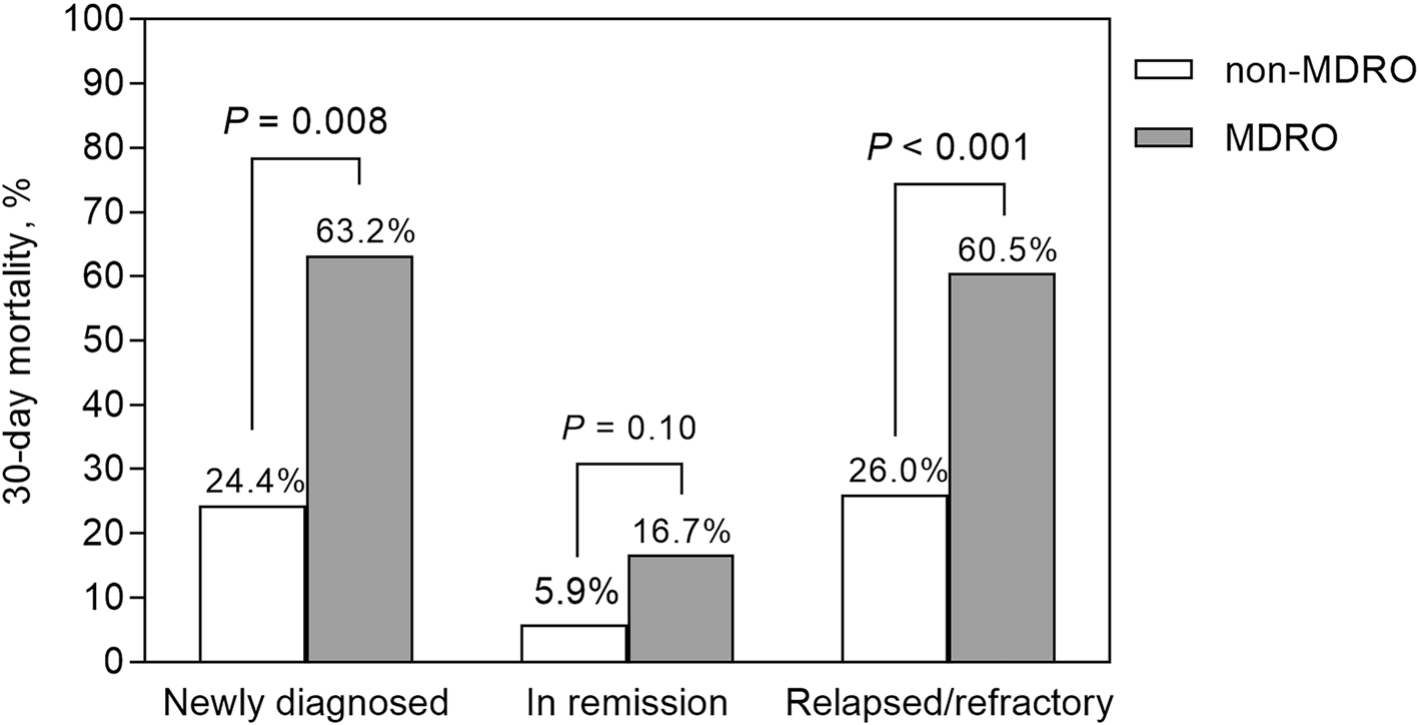
Comparison of 30-day mortality between patients infected with multidrug-resistant organisms (MDROs) and those infected with non-MDROs according to disease status. Among patients newly diagnosed with malignancy or experiencing relapsed/refractory disease, the mortality rate was higher in the MDROs group compared to the non-MDROs group. However, this difference was not observed in patients who were in complete remission.

**Figure 4:**
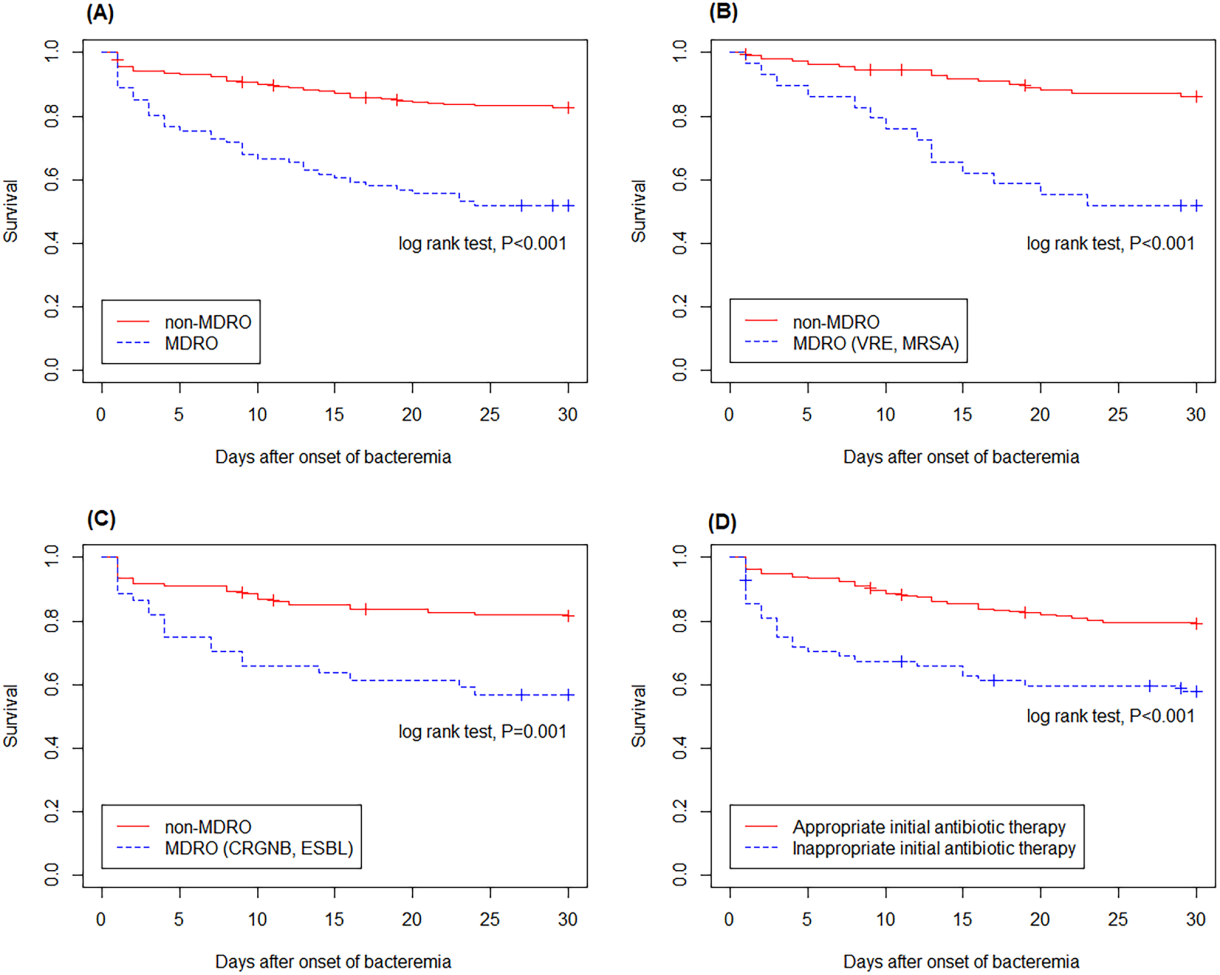
Kaplan–Meier analysis demonstrated a higher mortality rate among patients infected with multidrug-resistant organisms (MDROs) compared to those infected with non-MDROs (**A**). Similar trends were observed in patients infected with Gram-positive MDROs (**B**) and Gram-negative MDROs (**C**). Patients who received inappropriate empirical therapy exhibited a higher mortality rate compared to those who received appropriate empirical therapy (**D**).

Of the 328 patients with bacterial BSI, 82 (25.0%) died within 30 days of BSI onset (Table 2). The 30-day mortality rate differed according to disease status: 35.9% (23/64) for newly diagnosed cancer, 7.9% (10/126) for complete remission, and 35.5% (49/138) for relapsed/refractory disease cancer (*P* < 0.001) (Table 3). The mortality rate was higher in patients infected with MDROs than in those infected with non-MDROs (48.1 vs. 17.4%; *P* < 0.001). This finding was evident in patients who were first diagnosed with malignancy (63.2 vs. 24.4%; *P* = 0.008) and relapsed/refractory disease (60.5 vs. 26.0%; *P* < 0.001) but not in those who were in complete remission (16.7 vs. 5.9%; *P* = 0.10) (Fig. 3). Kaplan–Meier analysis showed that the mortality rate of patients infected with MDROs was higher than that of patients infected with non-MDROs (log-rank test, *P* < 0.001; Fig. 4A). Similar findings were observed in patients infected with Gram-positive and Gram-negative MDROs (Fig. 4B,C).

**Table 3:** Univariate and multivariate regression analyses of risk factors for 30-day mortality in 328 patients with hematological cancer and bacterial BSIs.

Characteristic	Survived (*n* = 246)	Deceased (*n* = 82)	Univariate Analysis		Multivariable analysis	
			OR (95% CI)	*P* Value	OR (95% CI)	*P* Value
Age ≥ 65 years	95 (38.6)	48 (58.5)	2.24 (1.35–3.75)	0.002	2.15 (1.12–4.23)	0.02
Male sex	128 (52.0)	41 (50.0)	0.92 (0.56–1.52)	0.75		
Underlying comorbidity						
Diabetes mellitus	51 (20.7)	16 (19.5)	0.93 (0.48–1.71)	0.81		
Cerebrovascular accident	11 (4.5)	6 (7.3)	1.69 (0.56–4.59)	0.32		
Liver cirrhosis	9 (3.7)	5 (6.1)	1.71 (0.51–5.11)	0.35		
Congestive heart failure	7 (2.8)	3 (3.7)	1.30 (0.27–4.79)	0.71		
Myocardial infarct	6 (2.4)	3 (3.7)	1.52 (0.31–5.90)	0.56		
End stage renal disease	2 (0.8)	3 (3.7)	4.63 (0.75–35.65)	0.10		
Chronic obstructive lung disease	5 (2.0)	0	NC	NC		
Charlson comorbidity score	2 (0–2)	2 (0–3)	1.15 (0.97–1.36)	0.10		
Underlying hematologic cancer						
Acute myeloid leukemia	94 (38.2)	29 (35.4)	0.88 (0.52–1.48)	0.65		
Acute lymphoid leukemia	20 (8.1)	6 (7.3)	0.89 (0.32–2.18)	0.81		
Lymphoma	60 (24.4)	22 (26.8)	1.14 (0.63–1.99)	0.66		
Multiple myeloma	48 (19.5)	14 (17.1)	0.85 (0.43–1.60)	0.63		
Myelodysplatic syndrome	20 (8.1)	7 (8.5)	1.05 (0.40–2.49)	0.91		
Chronic myeloid leukemia	2 (0.8)	1 (1.2)	1.51 (0.07–15.92)	0.74		
Chronic lymphoid leukemia	1 (0.4)	2 (2.4)	6.13 (0.58–132.83)	0.14		
Stage of hematological malignancy						
In complete remission	116 (47.1)	10 (12.2)	1	NA		
Newly diagnosed	41 (16.7)	23 (28.0)	6.51 (2.93–15.41)	< 0.001	5.80 (2.25–16.20)	< 0.001
Relapsed/refractory	89 (36.2)	49 (59.8)	6.39 (3.18–14.01)	< 0.001	6.89 (2.98–17.88)	< 0.001
HSCT at onset of BSI	7 (2.8)	0	NC	NC		
Post-HSCT	10 (4.1)	4 (4.9)	1.21 (0.32–3.73)	0.75		
Underlying conditions						
LOS until onset of bacteremia, days	14 (2–25)	19 (2–38)	1.01 (1.00–1.02)	0.054		
ICU stay at onset of bacteremia	1 (0.4)	4 (4.9)	NC	NC		
Presence of central venous catheters	194 (78.9)	54 (65.9)	0.52 (0.30–0.90)	0.02	0.64 (0.32–1.31)	0.22
Laboratory data						
WBC ≥ 15,000	8 (3.3)	9 (11.0)	3.67 (1.36–10.10)	0.01	2.21 (0.67–7.24)	0.19
ANC, /μL	93 (25–1898)	284 (33–2280)	1.00 (1.00–1.00)	0.87		
ANC < 500/μL	151 (61.4)	45 (54.9)	0.77 (0.46–1.27)	0.30		
ANC < 100/μL	125 (50.8)	36 (43.9)	0.76 (0.46–1.25)	0.28		
Platelet, × 10^3^/µL	42 (24–83)	31 (18–62)	1.00 (0.99–1.00)	0.11		
C-relative protein ≥ 20 mg/L^a^	36/226 (15.9)	34/79 (43.0)	3.99 (2.26–7.08)	< 0.001	3.35 (1.68–6.77)	< 0.001
Polymicrobial infection	7 (2.8)	11 (13.4)	5.29 (2.01–14.85)	< 0.001	3.68 (1.07–13.31)	0.04
MBI-LCBI	97 (39.4)	23 (28.0)	0.60 (0.34–1.02)	0.07		
Infections caused by MDROs	42 (17.1)	39 (47.6)	4.41 (2.56–7.64)	< 0.001	3.02 (1.52–6.04)	0.002
Inappropriate initial antibiotic treatment	40 (16.3)	28 (34.1)	2.67 (1.51–4.71)	< 0.001	2.38 (1.11–5.13)	0.03

The data are presented as no. (%) of patients or median (interquartile range), unless otherwise indicated.

ANC, absolute neutrophil count; BSI, bloodstream infection; CI, confidence interval; HSCT, hematopoietic stem cell transplantation; ICU, intensive care unit; LOS, length of stay; MBI-LCBI, mucosal barrier injury laboratory-confirmed bloodstream infection; MDROs, multidrug-resistant organisms; NA, not available; NC, not calculated; OR, odds ratio; WBC, white blood cell.

^a^Measured in 305 patients.

**Figure 5:**
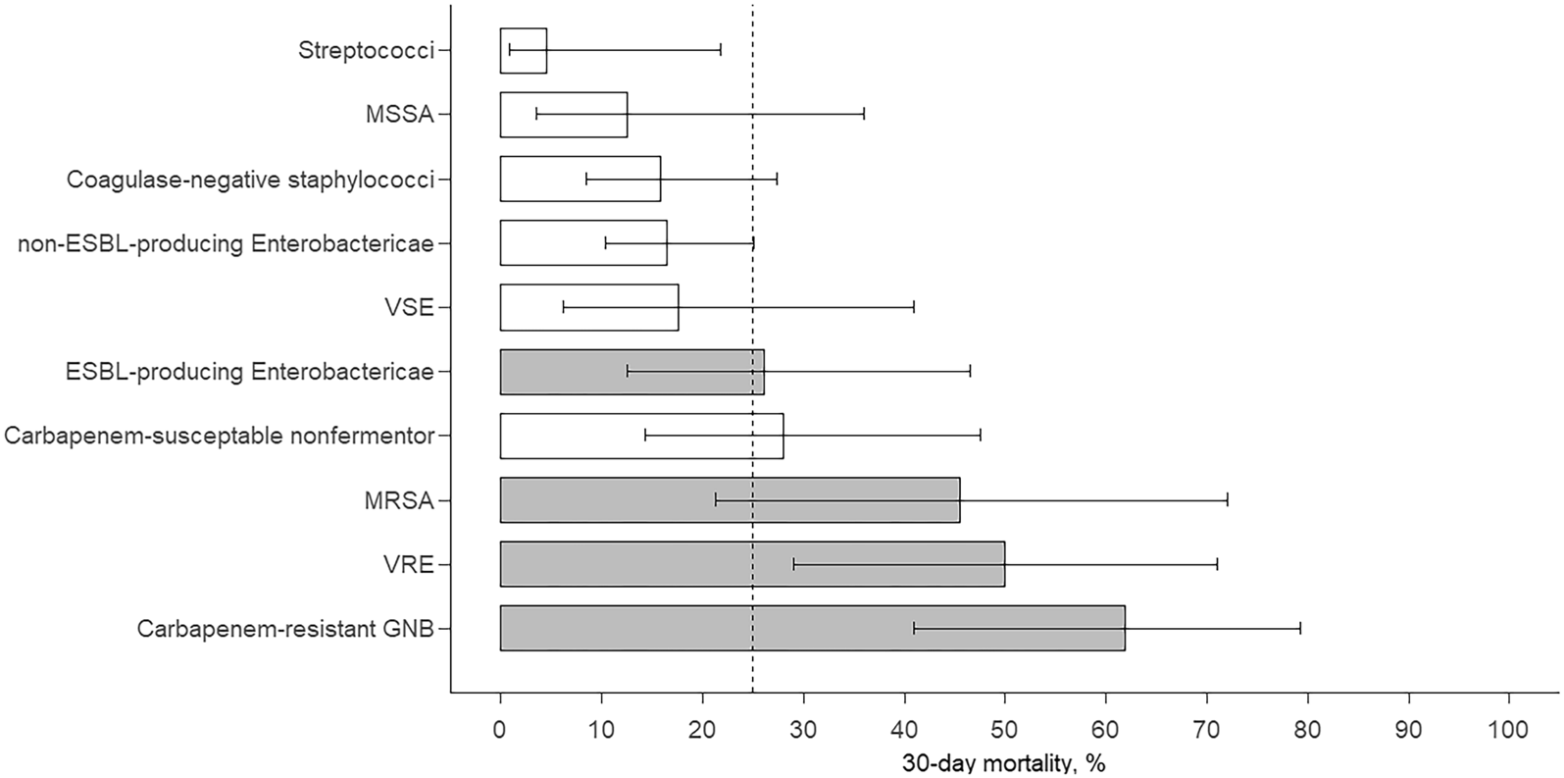
Bar chart showing 30-day mortality and 95% confidence intervals for BSIs caused by different pathogens. ESBL, extended-spectrum β-lactamase; GNB, Gram-negative bacteria; GPB, Gram-positive bacteria; MRSA, methicillin-resistant *Staphylococcus aureus*; MSSA, methicillin-susceptible *S. aureus*; VRE, vancomycin-resistant enterococci; VSE, vancomycin-susceptible enterococci.

Among the MDROs, carbapenem-resistant GNB exhibited the highest 30-day mortality rate (61.9%), followed by VRE (50.0%), MRSA (45.5%), and ESBL-producing *Enterobacteriaceae* (26.1%) (Fig. 5). The mortality for each pathogen, except for ESBL-producing *Enterobactericae*, did not differ between patients with and without neutropenia (Supplementary Table S1). The association between MDROs and 30-day mortality was evident for carbapenem-resistant GNB (odds ratio [OR], 5.61; *P* < 0.001) and VRE (OR, 3.25; *P* = 0.02) but was less evident for MRSA (OR, 2.60; *P* = 0.12) and not evident for ESBL-producing *Enterobacteriaceae* (OR, 1.06; *P* = 0.90).

Table 3 presents the results of univariate and multivariate analyses for 30-day mortality among hematological patients with bacterial BSIs. Univariate analysis indicated that age ≥ 65 years (*P* = 0.002), newly diagnosed disease (*P* < 0.001), relapsed/refractory disease (*P* < 0.001), absence of central venous catheters (*P* = 0.02), white blood cell ≥ 15,000/µL (*P* = 0.01), C-reactive protein level ≥ 20 mg/L (*P* < 0.001), infections caused by MDROs (*P* < 0.001), and inappropriate initial antibiotic treatment (*P* < 0.001) were the independent risk factors for 30-day mortality. Kaplan–Meier analysis showed that the mortality rate of patients who received inappropriate empirical therapy was higher than that of patients who received appropriate empirical therapy (log-rank test, *P* < 0.001; Fig. 4D). Multivariate analysis indicated that the independent risk factors for 30-day mortality were age ≥ 65 years (OR, 2.15; 95% CI 1.12–4.23), newly diagnosed disease (compared with complete remission, OR, 5.80; 95% CI 2.25–16.20), relapsed/refractory disease (compared with complete remission, OR, 6.89; 95% CI 2.98–17.88), C-reactive protein level ≥ 20 mg/L (OR, 3.35; 95% CI 1.68–6.77), polymicrobial infection (OR, 3.68; 95% CI 1.07–13.31), infections caused by MDROs (OR, 3.02; 95% CI 1.52–6.04), and inappropriate initial antibiotic therapy (OR, 2.38; 95% CI 1.11–5.13).

## Discussion

This study assessed trends in the prevalence of MDROs and their impact on outcomes in 328 hematological patients with bacterial BSIs. Over the 20-year study period, the rates of MDROs in bacterial BSIs within the population increased significantly by fourfold, affecting both Gram-positive and GNB. This substantial increase in MDR rates was also well-documented in a recent study involving 552 patients with hematological malignancies^10^. This study demonstrated that carbapenem resistance and MDR rates increased from 0 to 40% in *Pseudomonas* species and from 17 to 82% in *Acinetobacter baumannii*^10^.

In this study, the two notable factors for mortality in patients with hematologic cancer and bacterial BSIs were disease status and MDROs. We observed a higher mortality rate in patients with a newly diagnosed or refractory disease than in those in remission (Table 3). This increased mortality in patients in non-remission may be due to immunosuppression resulting from a higher disease burden. After adjusting for disease status, we found that MDROs were independently associated with a threefold increase in the odds of mortality (Table 3). The adverse effect of MDROs on patient outcomes was more pronounced in patients who were not in remission, with a mortality rate exceeding 60%, as opposed to 17% in those in remission (Fig. 3). Consistent with our findings, Scheich et al. found that the overall survival of hematological bacteremia patients infected with Gram-negative MDR bacteria was significantly lower than that of patients infected with Gram-negative non-MDR bacteria (85.6 vs. 55.9%)^11^. However, they did not find a significant association between the disease status and outcomes, potentially because of the small number of patients in remission (only three)^11^. In patients with hematological cancer, MDRO colonization is associated with subsequent bacterial BSIs^7,13^. Based on these findings and our data, we suggest that stringent infection control measures are critical in patients with hematological cancer to prevent MDRO colonization and subsequent BSI.

We assessed the effects of various pathogens on the patient outcomes (Fig. 5). Carbapenem-resistant GNB had the most adverse outcomes, whereas streptococci, coagulase-negative staphylococci, and non-ESBL-producing *Enterobacteriaceae* were associated with better outcomes. This finding aligns with recent observations by Weber et al., who analyzed 637 bacterial BSI episodes in patients with predominantly hematological malignancies^20^. Carbapenem-resistant GNB exhibited the highest mortality rate (62%), with a 5.6-fold increase in the odds of mortality in our study, consistent with the 9.5-fold increase in the hazard ratio for mortality reported by Weber et al.^20^. Notably, the CRE rate in this study was only 1.2%. Most carbapenem-resistant GNB were non-fermenting GNB, such as *A. bauamanni*, *P. aeruginosa,* and *S. maltophilia* (Table 1). Non-fermenting GNB are intrinsically resistant to many antimicrobials and can acquire resistance to any antimicrobial agents^8,14^. Bacterial BSIs caused by these organisms result in high mortality rates, ranging from 23 to 65% among patients with hematological cancer^21–23^. In a study by Scheich et al., patients with hematological bacteremia infected with MDR non-fermentators had worse overall survival than those infected with non-MDR non-fermentators (71 vs. 31%)^11^. VRE bacteremia resulted in a mortality rate of 50%, with a 3.2-fold increase in the odds of mortality compared to non-VRE bacteremia in our study, consistent with the 2.1-fold increase in the hazard ratio for mortality reported by Weber et al.^20^. Among patients undergoing hematopoietic cell transplantation, those with VRE BSI had a 4.7-fold increased risk of 1-year non-relapse mortality compared with patients without BSI^24^. The negative impact of polymicrobial BSIs in our patients is consistent with the findings of previous studies^5,7^.

Appropriate selection of empiric therapy is critical in hematologic patients at high risk of MDRO infection. In this study, a three-fold higher rate of inappropriate initial antibiotic therapy was observed in bacterial BSIs caused by MDROs than in those caused by non-MDROs (42 vs. 14%, respectively) (Table 2).We found that inappropriate initial therapy was independently associated with a 2.4-fold increase in the odds of mortality (Table 3). Our data are consistent with those of a study documenting the same association in patients with hematological malignancies and Gram-negative bacterial BSIs^18^. We observed that AML and more extended hospital stays were risk factors for MDROs (Table 1 and Fig. 2). In a previous study, 155 (50%) of 312 patients with AML were colonized with MDROs before, during, or after their hospital stay after induction chemotherapy^25^. The high rates of MDRO colonization in patients with AML may be attributed to prolonged hospital stays stemming from significant bone marrow suppression after chemotherapy. In our study, patients with AML had the most prolonged duration of hospitalization compared to those with other diseases. Therefore, in patients with AML or a prolonged length of stay, empiric antibiotic therapy against MDROs should be considered for suspected bacterial BSIs. However, it cannot be emphasized enough that the initial broad-spectrum antibiotic coverage for MDRO infections should be modified based on the final blood culture results.

The main limitation of this study was its retrospective nature, which could lead to potential missing or inaccurate data collection. However, we attempted to mitigate this issue by using a standardized data form for detailed clinical data collection and involving three authors (K. H. P., Y. J. J., and J. J. H.) in the data review. Disagreements among the authors were resolved through regular meetings. To ensure accurate identification of comorbid illnesses, we utilized both chart review and ICD-10 codes. Additionally, because the study was conducted at a single center, the generalizability of the findings to other settings with different patient populations or microbiological pathogens may be limited. Finally, the relatively small sample size compromised the statistical power of the study, potentially hindering the detection of differences within subgroups.

In conclusion, hematological patients with MDR bacterial BSI had a higher mortality rate than those with non-MDR bacterial BSI. The effect of MDROs on mortality varied considerably according to the type of disease status and causative pathogens but was independent of the type of hematological malignancy. Our findings underscore the importance of tailored antibiotic strategies and rigorous infection control measures to improve outcomes in patients with hematologic cancer.

### Supplementary Information

Supplementary Table S1.

## Data Availability

The datasets used and/or analyzed during the current study are available from the corresponding author on reasonable request.
