# Supplementary material for: Impact of multidrug resistance on outcomes in hematologic cancer patients with bacterial bloodstream infections
Source: Sci Rep. 2024 Jul 7;14:15622. doi: 10.1038/s41598-024-66524-w (PMC11228017; doi:10.1038/s41598-024-66524-w)
Supplement: Supplementary file 1 — Supplementary Table S1. [file 41598_2024_66524_MOESM1_ESM.doc]

**Supplementary Table S1.** Comparison of mortality rates for each pathogen between patients with and without neutropenia

| Organisms | All patients (*n* = 328) | Non-neutropenic patients (*n* = 132) | Neutropenic patients (*n* = 196) | *P*-value |
| --- | --- | --- | --- | --- |
| Gram-positive bacteria | 20.6 (29/141) | 23.5 (19/81) | 16.7 (10/60) | 0.44 |
| Methicillin-susceptible *S. aureus* | 12.5 (2/16) | 12.5 (1/8) | 12.5 (1/8) | 0.99 |
| Methicillin-resistant *S. aureus* | 45.5 (5/11) | 57.1 (4/7) | 25.0 (1/4) | 0.69 |
| Coagulase-negative staphylococci | 15.8 (9/57) | 20.5 (8/39) | 5.6 (1/18) | 0.29 |
| *Streptococcus* species^a^ | 4.5 (1/22) | 6.2 (1/16) | 0 (0/6) | 0.99 |
| Vancomycin-susceptible enterococci | 17.6 (3/17) | 40.0 (2/5) | 8.3 (1/12) | 0.39 |
| Vancomycin-resistant enterococci | 50.0 (9/18) | 50.0 (3/6) | 50.0 (6/12) | 0.99 |
| Gram-negative bacteria | 25.3 (42/166) | 31.1 (14/45) | 23.1 (28/121) | 0.40 |
| Non-ESBL producing *Enterobactericae* | 16.5 (16/97) | 16.7 (4/24) | 16.4 (12/73) | 0.99 |
| ESBL producing *Enterobactericae* | 26.1 (6/23) | 62.5 (5/8) | 6.7 (1/15) | 0.02 |
| Carbapenem-resistant *Enterobactericae* | 75.0 (3/4) | NA (0/0) | 75.0 (3/4) | NA |
| Carbapenem-susceptible non-fermenter^b^ | 28.0 (7/25) | 25.0 (2/8) | 29.4 (5/17) | 0.99 |
| Carbapenem-resistant non-fermenter^c^ | 58.8 (10/17) | 60.0 (3/5) | 58.3 (7/12) | 0.99 |
| Anaerobes | 0 (0/3) | NA (0/0) | 0 (0/3) | NA |
| Polymicrobials | 61.1 (11/18) | 66.7 (4/6) | 58.3 (7/12) | 0.99 |

The data are presented as (%) (number of deaths/total number of patients), unless otherwise indicated.

Abbreviations: NA, not available.

^a^ Included *Streptococcus pneumoniae* (*n* = 12), viridans streptococci (*n* = 9), and *Streptococcus agalactiae* (*n* = 1).

^b^ Included *Pseudomonas aeruginosa* (*n* = 20), *Acinetobacter baumannii* (*n* = 2), *Acinetobacter lwooffii* (*n* = 2), and *Sphingomonas paucimobilis* (*n* = 1).

^c^ Included *Pseudomonas aeruginosa* (*n* = 8), *Stenotrophomonas maltophilia* (*n* = 5), and *Acinetobacter baumannii* (*n* = 4).
